# Pharmacogenomics of pediatric asthma

**DOI:** 10.4103/0971-6866.73398

**Published:** 2010

**Authors:** Sarika Gupta, Shally Awasthi

**Affiliations:** Department of Pediatrics, C.S.M.M.U., Lucknow, Uttar Pradesh, India

**Keywords:** Asthma, pharamacogenomics, polymorphism, variability in response

## Abstract

**CONTEXT:**

Asthma is a complex disease with multiple genetic and environmental factors contributing to it. A component of this complexity is a highly variable response to pharmacological therapy. Pharmacogenomics is the study of the role of genetic determinants in the variable response to therapy. A number of examples of possible pharmacogenomic approaches that may prove of value in the management of asthma are discussed below.

**EVIDENCE ACQUISITION:**

A search of PubMed, Google scholar, E-Medicine, BMJ and Mbase was done using the key words “pharmacogenomics of asthma”, “pharmacogenomics of β-agonist, glucocorticoids, leukotriene modifiers, theophylline, muscarinic antagonists in asthma”.

**RESULTS:**

Presently, there are limited examples of gene polymorphism that can influence response to asthma therapy. Polymorphisms that alter response to asthma therapy include Arg16Gly, Gln27Glu, Thr164Ile for β-agonist receptor, polymorphism of glucocorticoid receptor gene, CRHR1 variants and polymorphism of LTC4S, ALOX5. Polymorphic variants of muscarinic receptors, PDE4 and CYP450 gene variants.

**CONCLUSION:**

It was concluded that genetic variation can improve the response to asthma therapy. However, no gene polymorphism has been associated with consistent results in different populations. Therefore, asthma pharmacogenomic studies in different populations with a large number of subjects are required to make possible tailoring the asthma therapy according to the genetic characteristic of individual patient.

## Introduction

Asthma is a chronic, inflammatory lung disease characterized by symptoms of cough, wheezing, dyspnea, and chest tightness, which occur in paroxysms, and is usually related to specific triggering events, airway narrowing that is partially or completely reversible and increased airways responsiveness to a variety of stimuli.[1]

The inflammatory features characteristic of asthma include infiltration of the airway by inflammatory cells, resulting in an increase in airway edema and mucus secretion, hypertrophy and hyperplasia of airway smooth muscle cells and increased airway vulnerability, all of which contribute to airflow obstruction.[2]

Asthma is the most common chronic disease in childhood in the first world countries. Data from the CDC-based National Centre for Health Statistics show an increase in asthma prevalence from 1980 to 1996 by greater than 50%.[3] The largest increase was seen in persons younger than 18 years. The CDC’s 2003 National Health Interview Survey yielded a lifetime asthma prevalence of 12.5% and current asthma prevalence of 8.5% among children ≤18 years.[4] Prevalence of asthma was 2.3 and 3.3% in the children of age group 6/7 and 13/14 years, respectively, in Lucknow, North India.[5] Asthma also negatively affects children during critical periods of growth and development, leading to increased annual cost of treating childhood asthma.[6]

Genetic and environmental factors are important in determining the risks of development of asthma. Childhood asthma is a disorder with genetic predisposition and a strong allergic component.

The goal of pharmacotherapy is to successfully maintain normal activity levels, including exercise, control chronic and nocturnal symptoms, optimize pulmonary function, prevent acute episode of asthma and avoid adverse effects of asthma medications. Medications used to treat asthma can be divided into two general groups: acute relief medications [short-acting β-agonists and systemic glucocorticoids (GC)] and chronic use medications [inhaled corticosteroids (ICSs), cromolyn/nedocromil, leukotriene modifiers, long-acting β-agonists, methylxanthines and omalizumab] [Table 1].[1]

**Table 1:** Drugs used in asthma therapy

Drug type	Mechanism of action	Examples
β-2 agonists	β-2 receptor stimulation → increased cAMP formation in bronchial muscle cells → relaxation	Albuterol
		Terbutaline
		Salmeterol
		Formoterol
Corticosteroids	Reduce bronchial hyper-reactivity, mucosal edema and suppress inflammatory response	Systemic corticosteroids
		Prednisolone
		Methyl prednisolone
		Hydrocortisone
		Inhaled corticosteroids
		Beclomethasone dipropionate
		Budesonide
		Fluticasone propionate
		Flunisolide
		Ciclesonide
Leukotriene modifiers	Leukotriene receptor antagonist antagonizes receptor mediated bronchoconstriction, increasing vascular permeability and recruitment of eosinophils	Montelukast
		Zafirlukast
	5-lipoxygenase inhibition → blocks LTC4/D4/B4 synthesis → prevents leukotriene induced response	Zileuton
Methylxanthines	Inhibition of phosphodiesterase	Theophylline
	Blockade of adenosine receptors	Doxophylline
Anticholinergics	Bronchodilation by blocking cholinergic constrictor tone	Ipratropium bromide
Nonsteroidal anti-inflammatory drugs	Inhibit allergen induced asthmatic responses and reduce exercise induced bronchospasm	Cromolyn
		Nedocromil
Anti-IgE antibody	Neutralization of free IgE in circulation	Omalizumab

As many as two-thirds of patients with asthma may not attain full control of their asthma.[7] Up to one-third of patients treated with ICSs may not achieve objective improvements in airway function or indices of airway reactivity.[8] Even more number of patients may not respond to leukotriene antagonists. One-third of patients using oral corticosteroids develop osteoporosis;[9] in addition, 3–5% of patients using a 5-lipoxygenase (ALOX-5) inhibitor develop increases in liver function enzymes.[10] A very small percentage of patients with asthma may be at risk of increased mortality with use of long-acting β-agonists. An estimated 70–80% of variability in individual responses to therapy may have a genetic basis.[11]

Pharmacogenomics defines the relationship between the variability in genetic code and the variability in responses to pharmacologic interventions. In addition, it offers to individualize treatment by using genetic information to improve drug efficacy and/or prevent side effects. Thus, the identification of genetic variants that identify asthmatic drug response will offer both prognostic assistance in the determination of response to existing therapy and the potential to develop novel pharmacologic agents. While there are a number of examples in which the approach is already in routine clinical usage, exploitation of this approach in asthma is still under development.

## Types of Genetic Variability

Single nucleotide polymorphisms (SNPs) are most commonly used to explore the pharmacogenetics of asthma.[12] Other types of DNA variation include deletions or insertions of one or more bases, variable number of tandem repeats (microsatellites) and varying combinations of SNPs and/or variable numbers of tandem repeats on a single chromosome (haplotypes).

## Factors Altering Predicted Pharmacogenomic Associations

It was observed that variations in genes having large effects on a pharmacologic response do not seem to produce an entirely uniform response to a drug because of additional sources of variability. This variation in response can be due to simultaneous genetic interactions, host factors and environmental effects.[13]

The simultaneous genetic alterations may enhance or detract from an examined pharmacogenomic effect. For example, if polymorphisms of the β-2 adrenergic receptor (β-*2AR*) predict diminished response to β-agonists in a patient, such patients simultaneously possessing a beneficial polymorphism in the corticosteroid pathway (e.g., corticotropin-releasing hormone receptor 1) may show an intermediate, rather than poor, response to a combination of long-acting β-agonists and ICSs. Host factors, which alter putative associations, are age, disease severity, concomitant drugs and disease etiology. Lastly, environmental factors can also alter the response. It was found that smoking modulates the response to ICSs and a recent study suggests that polymorphisms of the β-*2AR* are differentially associated with airway responsiveness in smoking versus non-smoking populations.

## Pharmacogenomics of Different Asthma Therapies

### β2 agonists

β-2 agonists are important bronchodilator drugs commonly used in the treatment of asthma. The β-*2AR* gene is expressed in bronchial smooth muscle cells and induces dilation in response to endogenous catecholamine or exogenous triggers. It is located on chromosome 5q31-32 and is highly polymorphic,[14] with three functionally relevant coding region polymorphisms (Arg 16 Gly, Gln 27 Glu, Thr 164 Ile) and multiple SNPs, either elsewhere in the coding region of the gene, in the 5' untranslated region or the 3' untranslated region of the gene, or in the adjacent genomic region. Martinez *et al*. found high linkage disequilibrium with the Arg-16 and Gln-27 alleles in asthmatic children, which makes it difficult to differentiate the effect of a single SNP because both SNPs are transmitted together.

The Ile-164 polymorphism, which is relatively uncommon, results in a substantial decrease in agonist binding affinity and coupling to adenylate cyclase. Polymorphisms Gly-16 and Glu-27 do not affect receptor binding or coupling, but markedly alter agonist-promoted receptor downregulation and functional desensitization; Gly-16 enhances this downregulation, whereas Glu-27 protects against it.

Earlier, it was found that homozygous Gly-16 was associated with a more severe asthma phenotype,[14] but this has not been supported by more recent studies.[15] Gly-16 has also been associated with nocturnal asthma[16] and in children it has been reported to be associated with decreased bronchodilator response to an inhaled β-2 agonist.[17] The Glu-27 polymorphism has been reported to be associated with decreased airway reactivity in asthma.[18] The Gln-27 allele, on the other hand, has been associated with elevated IgE levels and an increase in self-reported asthma in children.

Clinical studies have indicated that the Arg/Arg genotype for residue 16 of the β-*2AR* alters responses to treatment and disease severity in patients with asthma. Results from one study showed that albuterol-evoked Forced Expiratory Volume in 1 second (FEV1) was higher and the response was more rapid in Arg-16 homozygotes compared with carriers of the Gly-16 variant (18% increase vs. 4.9% increase, *P* < 0.03).[19] Similarly, spirometric assessment of 269 participants in a longitudinal study of asthma indicated that homozygotes for Arg-16 were 5.3 times more likely than Gly-16 homozygotes to respond (>15.3% increase in FEV1) to challenge with 180 mcg albuterol.[17] However, different pharmacogenomic associations were found in Indian and African-American populations.[20]

One study showed that patients with the Arg/Arg genotype had increased peak expiratory flow rates (PEFR) when β-2 agonists were withdrawn as a rescue inhaler. In contrast, patients with the Gly/Gly genotype showed good responses to β-2 agonist therapy, which reversed when it was withdrawn.[21]

One clinical trial results have indicated a decreased response to longer-term β-2 agonist treatment among patients with Arg/Arg genotype for residue 16 of the β-*2AR* as well as increased risk of exacerbations among patients with this genotype, who were treated with a short-acting β-2 agonist.[21,22] However, another study evaluating the effects of variation in the β-*2AR* gene on clinical response to salmeterol administered with fluticasone propionate found no variation in response to salmeterol after chronic dosing with an inhaled corticosteroid.[23] Recently, *ARG1* is identified as a new gene for acute bronchodilator response to β-2 agonist.[24]

Some of the studies assessed the association between β-*2AR* haplotypes and response, with inconsistent results. Drysdale *et al*. identified that haplotypes predict albuterol response.[20] Contrary to this, associations between β-*2AR* haplotype and drug response were not found in other studies.[20] In fact, Silverman and colleagues found an opposite association between albuterol response and haplotypes.[20]

To conclude, these data clearly indicate that genotype at the β-*2AR* can influence the response to a variety of treatment regimes with agents acting at this receptor. However, results from pharmacogenomic analysis of β-2 agonists have varied between different studies and populations [Table 2]. Therefore, additional researches are needed for confirmation of such studies and for treatment recommendations stratified by genotype.

**Table 2:** Pharmacogenomics of β-2 agonist

Study	Result	Population	References
Martinez *et al*.	Arg-16: better albuterol response	269 asthmatic children	17
Kotani *et al*.	Gly-16: lower albuterol response	117 Japanese asthmatics	20
Lima *et al*.	Arg-16: better albuterol response	16 Caucasian mild asthmatics	19
Israel *et al*.	For Arg-16, homozygotes using albuterol regularly had a decreased PEFR compared with those using as-needed albuterol	190 Caucasian asthmatics	21
Drysdale *et al*.	Albuterol response associated with haplotype pairs but not with single SNP	121 Caucasian asthmatics	20
	Haplotypes associated with better albuterol response in vivo is also associated with increased *β-2AR* gene and protein expression in vitro		
Taylor *et al*.	For Arg-16 homozygotes using albuterol regularly had major exacerbations	115 Caucasian mild asthmatics	22
Silverman *et al*.	SNP+523: better albuterol response association between albuterol response and haplotype pairs, opposite to the findings of of Drysdale *et al*.	707 Asthmatic children	20
Israel *et al*.	On scheduled albuterol, PEFR decreased in Arg-16 homozygotes, while it increased in Gly-16 homozygotes	78 Caucasian mild asthmatics	21
	On placebo, PEFR increased in Arg-16 homozygotes, while there was no change in Gly-16 homozygotes		
Taylor *et al*.	No association between *β-2AR* genotypes and haplotypes and albuterol response	176 Caucasian asthmatics	20
Kukreti *et al*.	Arg-16 homozygotes: lower albuterol response	80 Indian asthmatics	20
Woszczek *et al*.	Arg-16 homozygotes: better albuterol response	110 Polish asthmatics	20
	No association between *β-2AR* haplotypes and albuterol response		
Cho *et al*.	Genotypes and haplotypes with Arg-16: better albuterol response	195 Korean asthmatics children	20
Choudhry *et al*.	Genotypes and haplotypes with Arg-16: better albuterol response among	667 Latino family	20
	Puerto Rican but not Mexican asthmatics		
Tsai *et al*.	Cys-19: lower albuterol response	264 African asthmatics and 176 controls	20
Hawkins *et al*.	SNP+79 associated with albuterol response in African-Americans	560 White and African-American asthmatics and 625 controls	20
	No association between *β-2AR* haplotypes and albuterol response		
Ferdinands *et al*.	No significant association in Whites	189 White asthmatics and 63	20
	For Arg-16 homozygote African-Americans using albuterol regularly had a better lung function compared with the Gly-16 homozygotes	African-American asthmatics	
Bleecker *et al*.	No pharmacogenomic effect of *β-2AR* variation on response to long acting *β-2* agonists	Study 1 → 2250 asthmatics	23
		Study 2 → 405 asthmatics	
Litonjua *et al*.	*ARG1* → a novel bronchodilator response gene	Four asthma cohorts	24

## Glucocorticoids

Although GC are the mainstay of treatment for bronchial asthma, there has been increasing recognition of a group of asthmatic patients who do not appear to benefit from glucocorticoid therapy, i.e., the GC-resistant (GCR) asthmatic.

Corrigan has shown enhanced interleukin-2 (IL-2) and human leukocyte antigen (HLA-DR) receptor expression on peripheral T lymphocytes in GCR as opposed to GC sensitive (GCS) asthma. In addition, he has shown that T cell proliferation and the elaboration of interferon (IFN-γ) and IL-2 from mitogen-stimulated T lymphocytes were inhibited by dexamethasone in GCS, but not in GCR subjects. Leung has examined the effects of a 1-week course of prednisolone on bronchoalveolar (BAL) cells obtained from patients with GCR asthma. It was shown that GCR subjects had elevated cell numbers expressing IL-2 and IL-4 before prednisolone treatment as compared to the GCS subjects. In contrast to GCS subjects, prednisolone failed to suppress IL-4 and IL-5 expression in the GCR subjects. Therefore, the airway cells from patients with GCR compared with those from GCS asthma patients have different patterns of cytokine gene expression and distinct responses to GC therapy.[25]

Studies have shown that the glucocorticoid receptor (GR) in GCR asthmatics exhibits a lower interaction with activator protein-1 (AP-1), and this effect is accompanied by raised levels of AP-1.[26] Mathieu *et al*. have shown that overexpression of the *GR* gene in A549 human lung epithelial cells, using a *GR* expression vector, resulted in repression of both nuclear factor kappa-B (NF-κB) and AP-1 activities in the absence of drug.[27] This effect was increased following the addition of dexamethasone [Table 3]. AP-1 and NF-κB repression was also observed following overexpression of a *GR* ligand-binding mutant in the same cell line. This is in contrast to other studies carried out in different cells, which have shown *GR*-mediated AP-1 and NF-κB repression to be strictly hormone dependent.[28] These findings suggest that overexpression of the *GR* *in vivo* may be a potentially useful approach to gene therapy, especially as a complimentary or alternative treatment for GCR asthmatics.

**Table 3:** Pharmacogenomics of corticosteroid

Study	Result	Population
Tantisira *et al*.[32]	*CRHR1* variants → increased response to inhaled corticosteroids	1041 mild to moderate asthmatic children
Tantisira *et al*.[33]	*TXB21* variation → improved methacholine responsiveness	1041 mild to moderate asthmatic children

In addition, it was found that T lymphocytes and monocytes from GCR subjects generate a twofold excess of Fos protein which is secondary to an increase in the c-Fos transcription rate. Thus, excess c-Fos results in perpetuation of AP-1 mediated inflammation and renders the therapeutic effects of GC less effective by sequestration of GR within the nucleus.[29]

Many cases of GC resistance may be due to mutations or polymorphisms present in the *GR* gene (*GR/NR3C1*).[30] There are two naturally occurring isoforms of the *NR3C1*: *GR*-α (functional) and *GR*-β (no hormone-binding ability). Two types of GCR asthma have been identified: type I (>95% of cases) is cytokine induced and is associated with increased expression of *GR*-β and type II (<5% of cases) is due to low numbers of *GR*. Clinically, type I GCR asthmatic patients present with severe side effects. Type II GCR asthmatics have a generalized primary cortisol resistance and do not develop steroid-induced side effects.[31]

Tantisira and colleagues suggested a relationship between the response to GC and a polymorphism of Corticotrophin Releasing Hormone Receptor 1 (*CRHR1*) gene. In the individuals homozygous for the minor allele, the mean percentage change in FEV1 was 13.3% versus 5.5% for those homozygous for the wild-type allele.[32] Same workers had recently demonstrated that a nonsynonymous SNP in the *TBX21* (*H33Q*), the gene coding for transcription factor T-bet (T-box expressed in T cells), is associated with significant improvement in methacholine responsiveness in children with asthma. However, the minor allele frequency for this SNP was only 4.5%, and there were no homozygotes observed in more than 500 subjects, suggesting that although the effect of such a mutation may be large, it may only affect a small number of individuals.[33]

### 

#### Leukotriene modifiers

Leukotrienes, released from mast cells, eosinophils and other inflammatory cells in the airways of patients with asthma, are potent contributors to the physiological and pathological changes characteristic of asthma. Antileukotriene therapies inhibit synthesis of leukotrienes through *ALOX5* inhibition or by blocking the cysteinyl leukotriene receptor. Polymorphisms of the *ALOX5* promoter gene and the leukotriene C4 (*LTC4*) synthase gene have been associated with changes in function of these genes, leading to association studies of the polymorphisms’ effects on responses to leukotriene modifier therapy.

In 221 patients with mild-to-moderate asthma treated with an *ALOX5* inhibitor, ABT-761 (*n* = 114), or placebo (*n* = 107), 64 patients (among receiving active treatment) with at least one wild-type allele of the *ALOX5* promoter locus had greater improvement in FEV_1_ than the 10 patients without any wild-type alleles (18.8% improvement vs. 1.1% decline, *P* < 0.0001).[34] In a corroborating study involving the leukotriene receptor antagonist zafirlukast, patients with no wild-type alleles had a 2.3% decrease in FEV_1_, whereas 44 subjects with two wild-type alleles and 19 subjects with at least one wild-type allele had improvements in FEV_1_ of 9.1 and 12.8%, respectively.[35]

The *LTC4* synthase gene polymorphism has been correlated with the response of asthma patients to zafirlukast. Those with variant *LTC4* synthase genotype (C/C or C/A genotype, *n* = 13) had a 9% increase in FEV1, whereas patients with the wild type (A/A genotype, *n* = 10) had a 12% decrease.[36] This polymorphism also shows an association with aspirin induced asthma and also contributes to increased *LTC4* in the airway.[37] It is also considered as a potential risk factor for adverse reactions to nonsteroidal analgesics in asthma. The mechanism postulated is alteration of expression pattern of the enzyme [Table 4].

**Table 4:** Pharmacogenomics of leukotriene modifiers

Study	Result	Population
Drazen *et al*.[34]	*ALOX5* variation – increase in FEV1 with ABT-761	221 Moderate asthmatics
Sampson *et al*.[37]	*LTC4S* variation – increase in FEV1	23 Asthmatic patients
Anderson *et al*.[35]	*LTC4S* variation – increased response and FEV1 with zafirlukast	

#### Muscarinic receptors

Polymorphic variation within muscarinic M2 and M3 receptors could alter treatment responses to anticholinergic agents (ipratropium bromide). In Maltese asthmatic individuals, two degenerate polymorphisms in the coding region (1197T/C, Thr-Thr and 976A/C, Arg-Arg) and a common SNP in the 3' non-coding region (1696T/A), in M2 receptor gene have been identified, which are not relevant functionally.[38] However, no variation has been identified in the M3 coding sequence. In the Japanese population, a degenerate polymorphism in M2 coding region (1050A/G) and a degenerate M3 substitution (261C/T) in M3 coding region were identified.[39] Recently, a variable tandem repeat in the human muscarinic M2 gene promoter has been shown to influence gene transcription in cultured cells. It has been suggested that this variation may be contributory to the development of asthma symptom in patients and inter-individual variability in response to muscarinic antagonists.[40]

## Phosphodiesterase

The phosphodiesterase E4 (PDE4) represents the predominant cAMP hydrolyzing activity in human airway smooth muscle.[41] Increased activity of PDE4 is expected to decrease β-2 agonist response by degrading β-*2AR*. It is also expected to alter the response to theophylline; however, *in vivo* PD inhibitory action of theophylline is yet to be cleared.[42]

It has been suggested that *PD* genes contain a number of polymorphisms, however, presently no data are available on the mutation screening of *PD* gene in asthmatics.

### 

#### Other Genetic Factors

Antiasthma drugs, which are subject to CYP450 metabolism in humans, would be expected to display altered pharmacokinetic profiles in patients carrying the appropriate CYP450 gene variants. Montelukast is sulfoxidated and 21-hydroxylated by the CPY3A4 P450 isoform, while CYP2C9 mediates methyl-hydroxylation of the drug.[43] Salmeterol and budesonide are likewise oxidized by CYP3A,[44] while CYP1A2 is the major enzyme which metabolizes theophylline at therapeutic concentrations.[45] Functional polymorphisms affecting the genes coding for these CYP450 isoforms might be important determinants of responses to these drug treatments in asthmatics. Therefore, until further results are available, it can be postulated that CYP450 polymorphisms may contribute to variations in therapeutic responses to antiasthmatic drugs which are metabolized by the respective variant. Fast metabolizers may show decreased treatment responses, while slow metabolizers may be more likely to experience adverse effects.

Eotaxin Chemokine (C-C motif) ligand 11 is a potent eosinophil chemoattractant. Recent results have indicated that the genetic variation at the *CCL11* locus is an important determinant of serum total IgE levels among patients with asthma[46] and it is reasonable to suggest that *CCL11* genotype may influence the response to medications that exert their effect via IgE receptors (e.g., Omalizumab). However, studies carried out, to date, have not evaluated this possibility.

## Current and Future Perspectives of Pharmacogenomics

Pharmacogenomics enables a more patient-focused prescribing and helps to ensure that patients receive the drugs that would benefit them the most. Pharmacogenomic knowledge may also help to develop drugs that provide efficacy in a large number of patients or promote the development of new drugs specifically designed for pharmacogenomically compromised patients. At present, the currently available data regarding asthma pharmacogenomics may not be sufficient to justify routine genotyping of all patients prior to treatment. However, as new data become available and novel therapies are developed, the knowledge of patient’s genotype will be a necessary requisite in order to enable pharmaceutical companies and prescribers to optimize management of the disease. Further clinical and molecular work is needed to combine genetics, pharmacogenomics, accurate disease phenotyping and environmental exposures to build the foundation for personalized and predictive medicine. If successful, the resulting paradigm shift in medical practice will lead to improved clinical outcomes and decreased health care expenditures. The ultimate goal is to enable physicians to identify those at risk for asthma, intervene to prevent or attenuate the disease, and select the optimal medical regimen for each individual patient.

In conclusion, it seems that asthma pharmacogenomic studies need to be replicated in prospective clinical trials in different populations with a large number of subjects being genotyped. It is suggested that large clinical trials which are proposed for asthma drugs experimentation should include a pharmacogenomic study as well. Hence, it will be helpful if genetic material is obtained in all clinical trials and considered for prospective genotype-stratified clinical trials.
